# Towards a Smart Environment: Optimization of WLAN Technologies to Enable Concurrent Smart Services

**DOI:** 10.3390/s23052432

**Published:** 2023-02-22

**Authors:** Ali Mohd Ali, Mohammad R. Hassan, Ahmad al-Qerem, Ala Hamarsheh, Khalid Al-Qawasmi, Mohammad Aljaidi, Ahmed Abu-Khadrah, Omprakash Kaiwartya, Jaime Lloret

**Affiliations:** 1 Communications and Computer Engineering Department, Faculty of Engineering, Al-Ahliyya Amman University, Amman 19328, Jordan; 2 Computer Science Department, Faculty of Information Technology, Zarqa University, Zarqa 13110, Jordan; 3 Computer Systems Engineering, Faculty of Engineering, Arab American University, Jenin P.O Box 240, Palestine; 4 College of Computing & Informatics, Saudi Electronic University, Riyadh 11673, Saudi Arabia; 5 Department of Computer Science, Nottingham Trent University, Nottingham NG11 8NS, UK; 6 Instituto de Investigación para la Gestión Integrada de Zonas Costeras, Universitat Politècnica de València, Camino Vera s/n, 46022 Valencia, Spain

**Keywords:** smart environment, real-time applications, QoS performance analysis, IEEE technologies

## Abstract

In this research paper, the spatial distributions of five different services—Voice over Internet Protocol (VoIP), Video Conferencing (VC), Hypertext Transfer Protocol (HTTP), and Electronic Mail—are investigated using three different approaches: circular, random, and uniform approaches. The amount of each service varies from one to another. In certain distinct settings, which are collectively referred to as mixed applications, a variety of services are activated and configured at predetermined percentages. These services run simultaneously. Furthermore, this paper has established a new algorithm to assess both the real-time and best-effort services of the various IEEE 802.11 technologies, describing the best networking architecture as either a Basic Service Set (BSS), an Extended Service Set (ESS), or an Independent Basic Service Set (IBSS). Due to this fact, the purpose of our research is to provide the user or client with an analysis that suggests a suitable technology and network configuration without wasting resources on unnecessary technologies or requiring a complete re-setup. In this context, this paper presents a network prioritization framework for enabling smart environments to determine an appropriate WLAN standard or a combination of standards that best supports a specific set of smart network applications in a specified environment. A network QoS modeling technique for smart services has been derived for assessing best-effort HTTP and FTP, and the real-time performance of VoIP and VC services enabled via IEEE 802.11 protocols in order to discover more optimal network architecture. A number of IEEE 802.11 technologies have been ranked by using the proposed network optimization technique with separate case studies for the circular, random, and uniform geographical distributions of smart services. The performance of the proposed framework is validated using a realistic smart environment simulation setting, considering both real-time and best-effort services as case studies with a range of metrics related to smart environments.

## 1. Introduction

There has been a continuous increase in the use of networking technologies in newer application domains, such as smart homes, intelligent transport systems, online commerce, and medical domains, as a result of advances in communication and Internet technology. Wireless Local Area Networks (WLANs) and mobile networks have been the primary technologies for wireless communication. WLANs and mobile networks are becoming more common technologies for smart environments as they have more use cases and are easy to install while being relatively inexpensive. Wi-Fi networks employ a standard known as IEEE 802.11 as their Media Access Control (MAC) protocol. It also makes it possible for people who are thousands of kilometers apart to share information with one another in the form of papers, photographs, and movies across the globe. All of these services and applications can be carried out using a WLAN as a transmission channel. There is a large number of physical layer communication technologies to choose from, making it difficult to determine which one would provide the best performance for a given use case in a smart environment [1]. IEEE technology in smart industrial communication networks at its peak performance, in contrast to previous technologies, is not always guaranteed and should not be considered a default answer without confirmation from various types of studies that provide an in-depth investigation of these technologies [2,3]. In other words, determining the best way to employ IEEE technology in industrial communication networks is quite similar to determining the best way to use an older piece of technology. It is important to note that each IEEE 802.11 standard has a unique set of advantages and disadvantages. For example, 802.11a is less likely than 802.11b or 802.11g to cause radio frequency interference (RF). In densely populated locations, the preferable technique is 802.11b owing to its ability to provide interactive audio, video, and picture services. Although this is true, the range is inferior to that of 802.11b, and thus the two cannot be used in conjunction with one another. In light of this, the goal of this research is to determine which technologies and networks are most beneficial to end users and customers.

To enable smart environments, there are further considerations that need to be taken into account before picking a technology and network design that will be most effective when put into practice, including the number of access points, the number of nodes, and the type of data being communicated. QoS measures must therefore be used to ensure consumer satisfaction during selection. IEEE 802.11b/g/n and IEEE 802.11a are both wireless networking technologies; however, they operate in different frequency ranges. It is possible to use IEEE 802.11n to operate in the 5 GHz frequency spectrum if so desired. IEEE 802.11ac, on the other hand, can only operate at a frequency of 5 GHz because of technical constraints [4]. In order to use older hardware after a new software update, backward compatibility between IEEE 802.11 technology generations is needed. For the first time, new technologies can be implemented on a large scale because IEEE 802.11ac and 802.11n nodes are backward compatible. To put it another way, wireless AC (the router implementing the wireless networking protocol 802.11ac) can only be used to its full capacity when communicating from an IEEE 802.11ac device to another IEEE 802.11ac device, though nodes supporting all three standards can coexist in an 802.11 wireless LAN. Specifically, this is due to the router’s 802.11ac wireless networking technology. There will be limitations in terms of overall performance because of the previous standard. As a result, IEEE 802.11ac technology must be used by both the router and the devices. It was also found in [5] that the data rate performance of nodes in both the 802.11ac and IEEE 802.11a/n standards significantly decreased compared to a single network. Nodes from both sets of nodes were combined and simulated. Both sorts of nodes were shown to work together in this situation. As a result, it is critical to look for ways to improve the multistranded efficiency of IEEE 802.11 WLANs. Because we are looking for the finest technology and the best network design for a variety of technologies, we are interested in investigating mixed network topologies. Research on Internet apps as stand-alone services, in which every design and configuration is tailored to a single application, has now been expanded for mixed applications [6,7,8,9]. A wide range of services, as well as more nodes and IEEE technologies, are performed at specified percentages in these scenarios. In our previous work [6,7,8,9], we concentrated on the installation of Internet applications as an independent service. Configuration is used throughout this project. For each IEEE 802.11 standard, we studied the effects of node distribution (circular, random, or uniform) on network performance. Circular, random, and uniform node distribution all affected network performance; therefore, this study examined them all.

The management of this multiservice on wireless networks while maintaining QoS is already a significant difficulty; therefore, traffic measurements such as latency and jitter must be considered, acknowledged, and applied. Implementing QoS characteristics such as delay, jitter, and packet loss across real-time networks is likewise seen as a significant challenge. Choosing which technology to utilize and execute in a WLAN business from several physical layer technologies necessitates concurrent scientific analysis. On the other hand, it is now more challenging to decide which network configuration is ideal for allocating wireless network resources to deliver high quality due to the existence of three different network architectures, which include three tiers of service that have been referred to as the Basic Service Set (BSS), Independent Basic Service Set (IBSS), and Extended Service Set (ESS). In recent years, there has been an increase in high-quality digital content, as well as a change in end-user usage patterns. This fact, along with the adaptability, affordability, and digital media capabilities of the IEEE 802.11 standard, has caused Wi-Fi technology to dominate the market and created barriers to network efficiency and usability. The development of digital media distribution and streaming apps has been aided by the emergence of media platforms such as YouTube, Netflix, and others. If not appropriately handled, each of these services has a substantial influence on the level of consumer experience relating to data transfer rate, delay, and jitter [10]. The major contributions in this paper are listed below:

1. A network prioritization algorithm for enabling smart environments has been developed to determine an appropriate WLAN standard (or a combination of standards) that best supports a specific set of smart network applications in a specified environment.
2. A network QoS modeling technique for smart services has been derived for the assessment of best-effort HTTP and FTP and the real-time performance of VoIP and VC services enabled via IEEE 802.11 protocols in order to discover optimal network design structures.
3. A number of IEEE 802.11 technologies have been ranked through the use of the proposed network optimization technique with separate case studies for the circular, random, and uniform geographical distributions of smart services.
4. The performance of the proposed framework was validated using a realistic smart environment simulation setting that considered both real-time services and bad best-effort services as case studies, which included a range of metrics related to the smart environment.

In this paper, a novel algorithm was developed to compare the performance of the BSS, ESS, and IBSS nodes while providing best-effort services, such as HTTP and FTP, and real-time services, such as VoIP and VC, across various IEEE 802.11 technologies. The proposed algorithm will provide a ranking of the various IEEE 802.11 technologies. Further, this work provides its own case study of the analysis of these services for three spatial distributions (circular, random, uniform). In addition, we discuss how different geographical configurations influence the performance of each WLAN technology. This study considers various factors and provides the client with a menu of options. A compromise will have to be made between speed and cost. In many cases, the maximum data rate is unreasonably expensive for customers, so they should not be assumed to always be the best option. Clients are interested in seeing cost–performance data so they can choose a service with rates they are comfortable with at a price they are willing to pay.

This study’s novel contributions consist of (a) a framework/algorithm for analyzing network performance and (b) a method for implementing that analysis to determine the most effective network configuration, given the state of the art; additionally, the study aims to identify which IEEE technologies and network architectures can be used for future web-based programs and services. The performance of five distinct services (applications) was measured and analyzed in light of contextual variables such as geographic dispersion, network topology, and node density. Several quality of service (QoS) metrics were used and analyzed as part of the creation of a novel algorithm for comparing the performance of best-effort services, such as HTTP, FTP, and E-mail, to real-time services, such as VoIP and VC, across a number of different IEEE 802.11 technologies. These include latency, jitter, throughput, packet loss, download time, and page load time. The study’s overarching goal is to devise a weighted coefficient for each application’s metric parameters that can be used to rank the current IEEE 802.11 standards, using both stand-alone and mixed-use cases.

The purpose of this study is to identify which WLAN standard (or combination of standards) will provide the best overall performance for a certain smart environment scenario and for a set of applications. This research provides the consumer with a list of options after accepting a number of parameters. You might have to choose between speed and cost. This is not always the case because clients’ budgets might not allow for the fastest data rate. Customers want to know how much a service will cost them relative to how well it performs so that they are able to select a plan that offers the speeds they need at a cost they can afford. Because it infrequently corresponds to the actual delivered rates, the maximum data rate has little value to a prospective customer. Ordinary 802.11e is useless because no one ever achieves the theoretical maximum data rate (54 Mbps). It is not clear which network design is best for optimizing wireless network allocation and efficiency; furthermore, IBSS, BSS, and ESS have added to the uncertainty. This study examines the implications of varying the node count and the deployment of IEEE physical layer technologies across various spatial distributions. We want to highlight that four major types of topologies, including mesh, ring, uniform, and random topologies, were used to test network architecture and communication protocol performance for best-effort applications and real-time applications.

The rest of this article is divided into the following sections. Section 2 critically reviews the literature related to smart network environment prioritization. The proposed network selection algorithm for a mixture of services and related QoS derivations is presented in Section 3. The performance results are thoroughly reviewed and critically assessed in Section 4. The conclusion is presented in Section 5.

## 2. Related Work

The proposed method will be briefly compared to various algorithms in this section. The number of nodes, network architecture, IEEE standards, and simulation models for quality of service have all been contrasted and are reported in Table 1. The results for modern techniques [11,12,13] show the best network architecture based on metrics including throughput, jitter, and end-to-end latency, and deployed their models’ using nodes from (3, 9, and 18), (20), and (2). However, the validation of their suggested techniques has only been carried out using the BSS network design. The influence that the spatial distribution of nodes (namely circular, random, and uniform) has on the efficiency of a network has been investigated for each of the six IEEE 802.11 protocols. Recent works, such as the reviews in [14,15,16], have not demonstrated this distinct area of study. On the other hand, some studies were solely focused on evaluating methods for IEEE 802.11, 11ac, and 11n technologies, such as [17,18,19]. Moreover, [20,21] examined IEEE technologies with fixed node counts of (16) and (10), respectively. A related model with a Uniform Random Ordered Policy (UROP) was used to attain an energy harvesting efficiency as in [22], which presents a resolution to the problem of scheduling data broadcasts to take place in the wireless sensor networks of energy production systems; it was shown that UROP accomplishes the best possible fairness performance under a relatively common energy harvesting procedure over an unlimited time scale.

The WLAN 802.11 architecture is made up of various parts that work together to establish a connection to higher-level services. In IEEE 802.11 standards, waiting for medium access is one of the largest delays a node experiences. Unlike more modern IEEE 802.11 specifications, in which frames are transferred in bulk, traditional IEEE 802.11 specifications transfer frames individually, giving the node an opportunity to waste a considerable amount of time trying to reach the medium rather than actually transferring data. One simple approach to dealing with this issue is to deliver multiple frames together as a single aggregate frame [23]. The IEEE 802.11 MAC layer specifies two medium access coordination functions—the required DCF and the discretionary PCF. Asynchronous and synchronous transmissions are both possible in 802.11’s access functions. All 802.11 stations are required to use DCF because of the asynchronous transmission it enables. The PCF offers a synchronous service that, in essence, implements polling-based access [24]. The primary goal of both of the updated IEEE 802.11e and IEEE 802.11n protocols was to boost the efficiency of the MAC layer when transmitting video data. IEEE 802.11e defines a new Distributed Coordination Function, known as EDCA, to minimize transmission latency for a group of high-priority video streams over a shared channel, while IEEE802.11n defines modern aggregation, block acknowledgement, and reverse direction improvements for high-throughput WLAN transmissions [25].

The Base Station Switch (BSS) is the central component of an 802.11 WLAN. A Base Station System (BSS) is a collection of base stations in a wireless network that are coordinated using either a Distributed Coordination Function (DCF) or a Points Coordination Function (PCF). Yet, the transmission medium degrades as a result of interference from neighboring stations sharing the same physical layer, making some stations seem “hidden” from others. Services such as data delivery, authentication, and confidentiality are all provided by a station in a wireless LAN. Ad hoc networking, or IEEE 802.11 IBSS, is the official name. All stations can have direct conversations with any other BSS station without resorting to AP transmission. An ESS is a collection of BSSs used for infrastructure purposes. Networks in the backbone must be constructed with APs that control the flow of data in transmission. The MAC Service Data Units (MSDUs) are transported via the Distribution System (DS), which is also the backbone of the wireless network and may be responsible for the installation of both wireless and wired networks. Digital Submarine (DS) signals carry data from one ESS access point to the next [26]. There are M primary users (transmitters), M secondary users (receivers), and K secondary receivers and channels in the work of [27], which deals with vehicle networks aided by cognitive radio. Moreover, a channel is allocated for primary user data transmission when a request for use is received. The receiver has a data backlog and does not know the channel state or the statistics of the channel’s evolution; therefore, for each time slot, it picks a channel from K to M at random. Uniform Random Ordered Policy (UROP) is also introduced and shown to produce near-optimal throughput for a generic channel evolution process under the block fading assumption in this research.

Technology is improving the functionality of portable devices such as laptops and mobile phones, which allow users to access the internet and make calls when they are on the move. Self-organizing networks will make it easier for people to connect in the future by reducing the cost of communication [28] and simplifying the process of putting together and configuring a wireless network by eliminating the need for preexisting infrastructure. The use of an ad hoc network is suitable if you need to transfer a lot of data quickly from one device to another. There are few restrictions on where an ad hoc network can be set up. For this reason, they could be useful in a variety of settings, including commercial and non-profit enterprises, as well as for personal use at home. As a result, it is less complicated to use and costs less money for businesses [29].

Many issues arise because people are unable to properly configure a network’s topology. A comparison between two extremes can show how similar or different they are. There is no established policy for this network [30].

A shift is occurring from the traditional desktop computing environment, in which workstations connect through shared servers on a single network, to one in which many different platforms communicate over many different networks [31]. Multiple network communication describes this scenario. There has been rapid acceleration in this shift.

It evolves and modifies itself to meet the requirements of mobile workers and their teams. The next generation of wireless communication systems will need to accommodate a large number of autonomous mobile users rapidly. In a MANET, all of the nodes in the network structure communicate with one another without any central authority. One alternative is for every node to serve as a router [32].

In contrast to the limitations previously described, this study demonstrates the implementation of a novel parametric assessment method capable of determining the optimal network configuration through the use of three distinct network architectures: one or more access points; an ESS/BSS and the non-availability of access points; and an ad hoc–IBSS architecture. The proposed method has been evaluated in accordance with the requirements of a total of six distinct IEEE standards for technical advancement, specifically 802.11, 11a, 11b, 11g, and 11n, for a total of five mixed-based applications with a range of different node sizes (1 to 65).

## 3. Network Prioritization for Smart Environments

The proposed network prioritization framework is a type of smart environment recommendation system that determines an appropriate WLAN standard or a combination of standards to best support a specific set of smart network applications in a specified environment. A network QoS modeling technique for smart services has been derived for the assessment of best-effort HTTP and FTP and the real-time performance of VoIP and VC services enabled via IEEE 802.11 protocols in order to discover optimal network architectures. Modeling, simulation, and experimentation are the three primary methods used in WLAN performance evaluation and network prioritization. It should be obvious that there are essential trade-offs, whereby it is truly crucial to each methodology to choose the right one for a given problem. Most of the time, the main goal is to enhance the key performance method and the strategic objectives that must be met. Instead of dissecting each strategy independently, we focus on the three trade-offs that are inevitable with any approach. When these concessions are carefully examined, an effective evaluation method whose implementation may be quite clear and obvious emerges.

Analytical or mathematical closed-form solution models are expected to provide a certain range of hypotheses for streamlining a system because the solution to the equations used to define the changes in the system is known. In addition, it is important to use a model faithfully by making sure that the model’s accuracy and reliability are as crucial as the intended application demands. The simulation could be interpreted as a highly specific and fully automated model. Because they often only model subsets of the actual performance of the network, but are so detailed that they are essentially equivalent to experimental research, they fall into a middle ground between mathematical/analytical models and experiments. When comparing practical application to theoretical and experimental endeavors however, its utility as an evaluation tool for the former stands out. In recent years, IEEE 802.11 technologies have become more widely available and inexpensive, ushering in a more favorable epoch for experimental WLAN measurement. Recently, testbeds have become widespread and are being implemented in a wide range of settings. Considering that nothing is ever relevant to the actual program/scheme except the program/scheme on its own, it may be less common to challenge the fidelity of experiments. Time and money are the usual costs that are considered when evaluating different methods of performance review. Analytical modeling is best avoided in situations where quick answers are required because of the time and expertise required to build it. However, once a model is fully developed, it is usually much quicker at achieving performance than experiments or simulations. Furthermore, analytical models can be developed in completely open-source environments for next to no additional cost. Learning to use a simulation tool can take some time, but there are many free options for wireless simulation. While the price of hardware is comparable between analytical models and simulation configurations, the benefit of the latter is that the code that recreates the performance of the system has already been generated. However, depending on the complexity of the simulated network, the simulation runtime may be prohibitively long if parallelization is not an option. As the necessary facilities are likely to be quite pricey and the knowledge required to adequately design and implement experimental work takes a considerable amount of time to develop compared to mathematical models or simulation platforms, experimentation is traditionally the most costly technique.

When it comes to large-scale applications and scalability, simulation and analytical models take precedence over experimental work. In practice, only minor tweaks to the code are needed to simulate a network with hundreds of access points. It is important to remember that simulating and modeling on a massive scale will take more time and, probably, a lot of technical manpower. In the end, there are advantages and appropriate applications for all methods of performance evaluation. Network simulation and modeling are commonly used when a fast and low-cost result is required. Experimentation appears to always be the best option when it is critical to keep production linked and close to a practical wireless network. Additionally, all methods have one thing in common: they produce a number of useful and fortunate results.

Due to the diversity in evaluation methods, we have opted to construct a full suite of WLAN system simulations, which grants us great adaptability and allows us to scale the framework more effectively and cheaply. The works cited in this research offered a more comprehensive analysis of the system as a whole. As a result, rather than modeling the processes within each node separately, we decided to model and simulate the network as a group of nodes for the three different network configurations. Further, our method’s distinctive feature is that it makes use of Riverbed’s extensive standard model library to support a variety of network models, protocol configurations, and geographic distributions. Through its Rapid Configuration features, the Riverbed Academic Platform library incorporates the distribution patterns for three spatial distributions (circular, random, uniform) and Riverbed (OPNET), and automatically builds the necessary distribution from its C or C++ source code based on user requirements. To accomplish our goal of contributing an answer to the question, “What WLAN standard (or mix of standards) will result in the best overall performance for a given mix of applications in a given environment?”, we chose a wireless protocol that meets user needs without any outside influence and developed a suite of system simulations. Furthermore, we made up a coefficient of importance for each QoS parameter used by each application. Five applications (VoIP, VC, HTTP, FTP, E-mail) were configured as mixing services and five mixed percentages were introduced that covered almost all distribution options for these services. Six IEEE technologies (11, 11a, 11b, 11g, 11e, 11n) were supported by OPNET academic licenses, with three network configurations (BSS, ESS, IBSS). All scenarios were run in all possible spatial distributions. The aforementioned conditions were tested across five distinct sets of nodes. Here, we want to highlight that we have presented a critical investigation of different network architectures that considers a range of communication protocols with the aim of enabling smart environments that focus on minimal jitter and higher throughput performance. The purpose and requirement of this study is to establish which network architecture is most suited for each of the five distinct mixed-use case studies that have been considered in this paper.

### 3.1. System Model and Preliminaries of Smart Environments

Wireless communication technologies require only a modest amount of cable infrastructure, making them an extremely efficient and cost-effective method for linking network nodes. Mobile networks are essential to the operation of a wide variety of applications, including C-ITS (Cooperative Intelligent Transportation Systems), automotive networks, precision farming with linked engines, and a large variety of functions available on smartphones. These technological advancements make it possible for applications to perform the purposes for which they were designed. The great majority of these applications depend on mobile nodes in order to achieve the maximum throughput that is possible for them. In order to obtain the greatest potential throughput, the communication equipment needs to be capable of achieving the highest feasible physical data rate. WLANs that are based on the IEEE 802.11 standard do away with the requirement for cables or mobility in public locations such as airports and workplaces [33]. WLANs are also essential due to the simplicity with which they may be installed and the rapidity with which they can transfer data. This section focuses on IEEE network infrastructure.

The streaming of live video, social networking, or the playing of online games can all benefit from Wi-Fi. High-quality video over WLANs [34] is still challenging to send due to bandwidth restrictions. Wireless communication has become an essential component of modern life as a result of rapid advancements in wireless technology and the increasing need for people to always be connected. In recent years, the amount of high-quality digital information that consumers have access to has grown, as has the method by which they consume it. The dominance of Wi-Fi in the market is due to a variety of causes, including a lack of competition. The standard’s adaptability, affordability, and support for digital media are just a few of the many benefits it offers. As a result, network efficiency and usefulness have been hindered. Digital media delivery and streaming application growth have been spurred by the emergence of media platforms such as YouTube and Netflix among many others. Due to their impact on latency, jitter, and throughput for end users, these applications must be taken into consideration while designing networks.

The features of quality of service measurements are used to define performance metrics. As stated in Table 2, which describes the primary QoS expectations and standards for each application [35], the acceptable threshold for each QoS metric parameter may be found in the table (traffic to be carried by the bearer).

The following quality of service measures have a direct influence on the overall quality of applications:

- Latency: the amount of time measured in seconds that it takes for data or voice traffic to travel from node A to node B over the network.
- Jitter (sec) is a variation in latency that is caused by queuing.
- Throughput: the rate at which data packets are sent from one point to another during a specific amount of time, which is measured in bits per second.
- Packet loss: refers to the percentage of packets that are lost along the transmission channel after the user has already transmitted the packet across the network.
- An important coefficient, abbreviated as ICP, is assigned to each application parameter in accordance with the effect that the parameter will have on the data quality provided by the service. The threshold values that are presented in Table 2 reflect the importance of every QoS parameter regarding the overall quality of each application. In order for these qualitative characteristics to be taken into account in a simulation, there must be a numerical representation of them (H = 1, M = 0.5, L = 0.01, and VL = 0).

### 3.2. Smart Environment Prioritization

**Figure 1:**
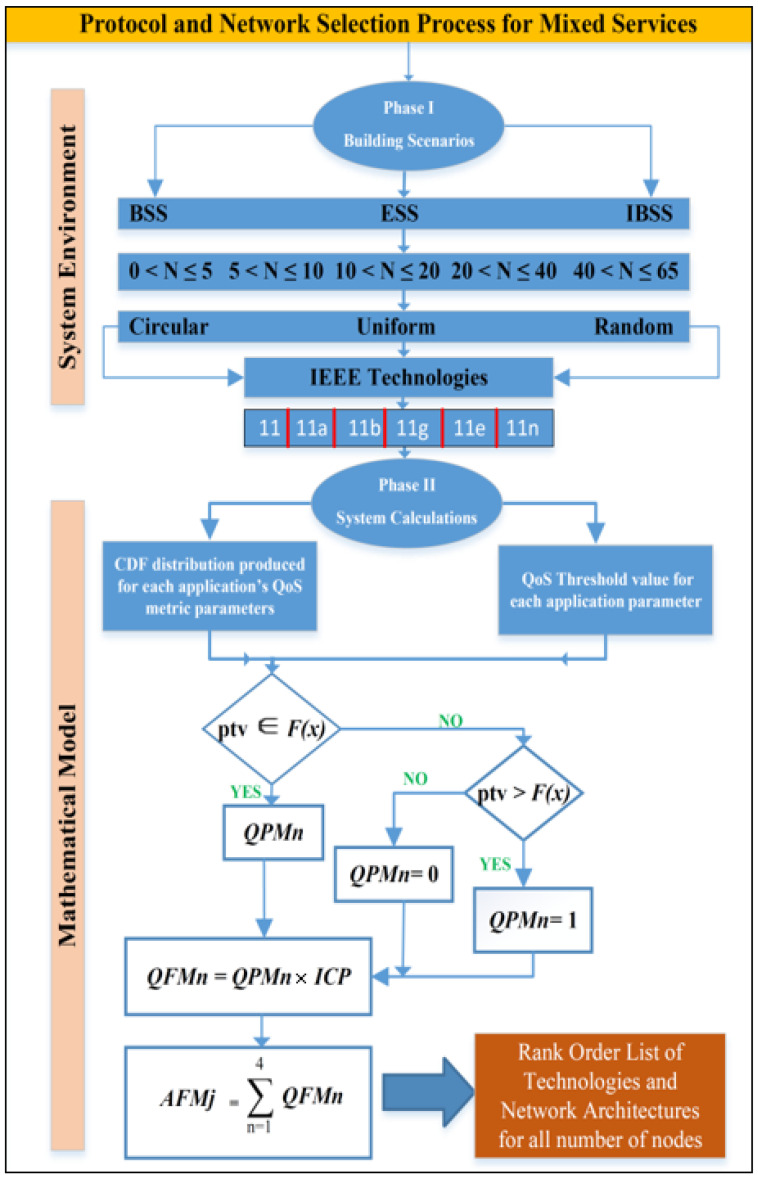
Flow diagram of the proposed algorithm in terms of both system environment and mathematical model.

Smart environment prioritization is used in this study to build and explore several smart application scenarios using an OPNET simulation platform called Riverbed Modeler 17.5 [36]. Thanks to Modeler’s ease of use and scalability, it is now able to research communications networks, network equipment, business applications, services, and protocols. Technical companies who have been most successful in their R&D efforts have followed the approach shown below. OPNET was used to simulate the procedure, and the following two essential source inputs were considered. User and technical requirements (standards) can be set up in several ways. Here, you will find an explanation for each of these elements, as shown in Figure 1.

Configurations for users (clients) can include a wide range of options, including but not limited to the following options:

- The total number of network nodes that must be present (where % is the total number of nodes in each application under consideration). The following research study used mixed applications:
  1. A mixture of 50% VoIP and 50% VC (real-time applications).
  2. E-mail (30%), FTP (30%), and HTTP (40%) (best-effort applications).
- Circular (oval), uniform (grid), or random topologies can be defined for these nodes using a spatial distribution.
- Technical Specifications.

**Figure 2:**
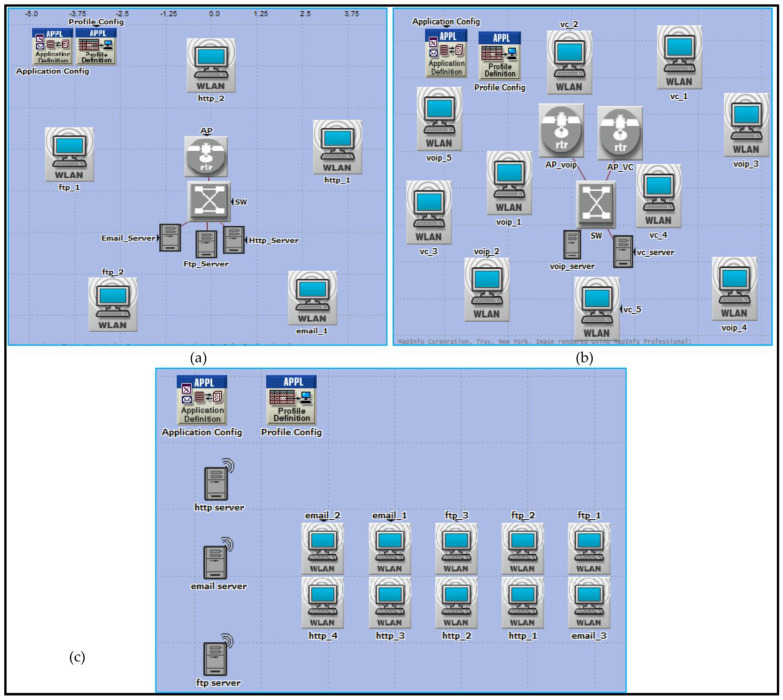
Design of the three network architectures across three spatial distributions for service mixing: (**a**) Basic Service Set (BSS), (**b**) Extended Service Set (ESS), and (**c**) Independent Basic Service Set (IBSS).

It is possible to use the physical layer specifications to create a framework for many different design scenarios. Networks indicate communication between multiple wireless components in one of two ways: either without an access point (ad hoc) or with an access point present (Wi-Fi) (BSS and ESS), as shown in Figure 2. This network’s nodes, which are divided into five distinct divisions, are essential (0–5, 6–10, 11–20, 21–40, and 41–65).

It is in accordance with the literature [20,37,38] that nodes are considered. To preserve network performance quality with these five groupings of nodes however, all observed outcomes are acceptable. These restrictions are due to the fixed capacity of bandwidth in a network; if the network has a large number of nodes, a modest amount of traffic can cause performance degradation. Because there are more nodes in the network, this is the case. This has only been tested in spaces between 2 and 3 m and 10 and 14 m because it is the normal size of a laboratory in a university, college, or school. An OPNET simulator was used to evaluate performance in a wide range of use cases for each application. There are other examples of these outcomes that are displayed in Figure 2a–c. The 802.11 (FHSS), 802.11a (OFDM), 802.11b (DSSS), 802.11g (OFDM), and 802.11n MAC layer technologies were used in this research (MIMO-OFDM). The 802.11e standard also enables contention-free bursts (CFBs) and defines quality of service (QoS) for 802.11. Because of CFBs, many frames can be sent at once if the transmission opportunity (TXOP) granted to a station is sufficient for this. Quality of service enabled access points (QAP) define hybrid coordinators, which feature an EDCA (Enhanced Distributed Channel Access) access mode. It is analogous to DCF, yet assigns varying weights to various services (such as DiffServ). IEEE 802.11e must be implemented in both the access point (AP) and the station (STA). The ability to work with STA devices that do not support QoS or 802.11e is also a benefit. VoIP traffic was configured for real-time applications with the following parameters during the simulation’s 20 min runtime: G.711 encoding technique, one voice frame per voice packet, and interactive voice communication, which are all features of the G.711 standard. In addition, the frame inter-arrival time was 15 frames per second, and frame size was 128 × 240/128 × 240 pixels (bytes). Files up to 50 KB can be transferred through FTP, whereas E-mail files can be up to 20 KB.

The customer is presented with a selection of options based on a variety of factors. There may be a trade-off between the expense and the speed of the vehicle. Since doing so may be prohibitively expensive for them, it is not true that customers will always opt for the fastest data rate available. A cost–performance comparison is what they are after so that they can find a service with the speeds they are willing to put up with for a price they can afford. It would be useless to provide a potential customer with a maximum data rate because it is merely a theoretical figure and frequently does not reflect the actual delivery rate. Since the theoretical data rate is so high, why do we not use it? The theoretical maximum speed of 802.11e is 54 Mbps; however, in practice, no one even comes near this [39]. Because the system handles both uplink and downlink applications, the minimum data rate shown in Table 3 is due to the fact that the theoretical upper bound of the system’s uplink performance is very close to the minimum data rate mandated by the reference architecture, which is thus more likely to help users by being more realistic [40]. In light of the fact that the system manages both downlink and uplink application tasks, a minimum data rate is more likely to benefit consumers while also being more feasible.

### 3.3. QoS Derivation for Smart Environments and Services

The mathematical model and system computations are depicted in the lower part of Figure 1, which represents Phase II. The CDF distribution and the QoS threshold values for each application are the mathematical inputs that are used by the algorithm. The results of the literature review are shown in Table 2 [41]. After each of the simulation scenarios was completed, the CDF distribution for the QoS metric parameters derived from OPNET was then created. The use of mathematical computations was required in order to determine whether or not a certain circumstance satisfied a number of crucial parameters for each application. The computations that were performed using this method will be discussed in the following parts, beginning with the results of each of the aforementioned projects.

**Figure 3:**
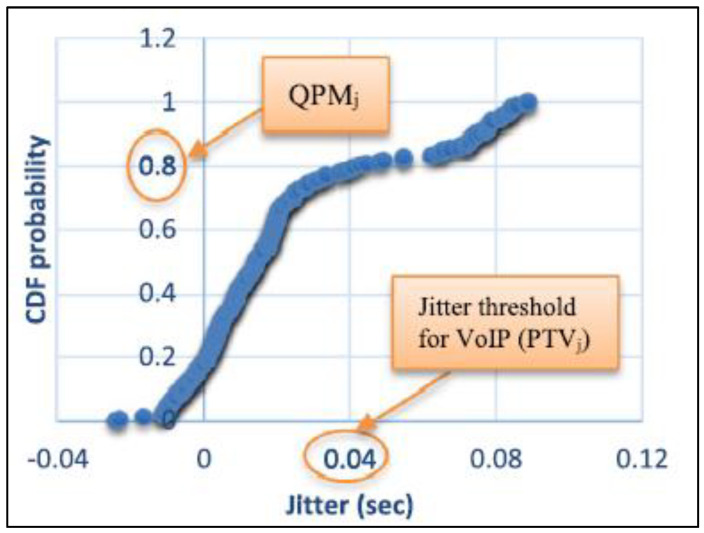
QPM for jitter.

QoS Performance Metric (QPM): the value that is generated by utilizing the parameter threshold value (PTV) as an application quality of service metric when defined in the cumulative distribution function (CDF), with distribution F(n) given by Equation (1), as illustrated in Figure 3, for each QoS performance criterion n.

(1) $${\mathrm{QPM}}_{n}=F\left(\mathrm{ptv}\right)$$

The value created by applying a weighting to the QPM (given by importance) is known as the QoS Fitness Metric (QFM), and it is specified by Equation (2) for each QoS metric parameter.

(2) $${\mathrm{QFM}}_{n}={\mathrm{QPM}}_{n}\times \mathrm{ICP}$$

When the QFM is equal to 0.8 and weighted by 1, it generates 0.8, which is the performance measure for jitter for the same QoS parameter. The coefficient of importance is high (H = 1) for this parameter. As a result, the coefficient of importance (H = 1) is multiplied by 80% of adequacy.

The final phase involves accumulating all QFMs for n application QoS metrics (throughput, delay, packet loss, and jitter) for each IEEE 802.11 standard g, with M denoting the machine-specific percentages in the mixed services scenarios, as shown by Equation (3).

(3) $${\mathrm{AFM}}_{j}=\sum_{n=1}^{4}{\mathrm{QFM}}_{n}$$

Each of the three network designs will have a ranking for the six technologies based on the AFMs of IEEE 802.11 technology. Ideal network architecture performance is then established for every node grouping. Figure 1 depicts the mathematical formulas used to determine the AFM value for each IEEE MAC technology. OPNET Modeler’s QoS metric settings, as well as the CDF distribution F(n) [41], will be provided and evaluated using the PTV in the following ways: For this metric parameter, the PTV has a CDF distribution that is identical to QPM if PTV F(n): ICP uses QPM as a weighting factor while generating QFM. The AFM is then created by combining all QFMs and is used to categorize IEEE technologies, as seen here. This signifies that the QFM has expanded and the QPM value has reached 1 if PTV > F(n). A value of 0 for QPM and QFM will be the result if PTV > F(n). The preceding sections have described how to determine QoS metric parameters for all applications, except the one that accounts for packet loss. OPNET Modeler’s packet loss parameter returns a Boolean value (0.0 or 1.0) that indicates whether or not a packet was accepted or rejected. However, a precise count of the number of packets lost is required for this investigation. Loss rate for an application packet is represented by $\omega_{i}$ on a node i. Equation (4) shows the proportion of discarded data packets (ki) to the total number of data packets ($\rho_{i}$) multiplied by 100%. A MATLAB tool was developed to calculate the percentage for all mixed applications. Packet loss percentages for hybrid applications and IEEE technologies are readily available within the OPNET Modeler.

(4) $$\omega_{i}=\left(\;\mathrm{ki}/\rho_{i}\right)\times 100\%$$

Utilizing the OPNET Modeler, traffic data are required in order to obtain information regarding the total number of packets that have been transmitted and received. The flowchart that was discussed before needs to be used to calculate the values of QPMs, QFMs, and AFMs. In order to do this, an exact packet loss ratio needs to be generated and presented in a CDF diagram. In order to determine which IEEE technologies are best suited for each application, each application’s set of QoS metric parameters must be generated for all possible combinations of network architecture and the three possible locational distributions. The nodes in each of the three spatial distributions must then be divided into five equal groups. This was achieved by grouping the nodes into groups across all three spatial distributions. In light of this, it is generally accepted that the statistical correlations between parameters (which establish thresholds) need to be taken into consideration in order to guarantee that all applications included in the mix can be simulated. By adopting these methodologies and taking into account the individual statistics of the parameters (as opposed to the joint statistics), one can get a comparative gauge for overall performance that is both useful and informative.

All the scenarios were developed, configured, performed, and analyzed using OPNET Modeler. For a 50% VC implementation, you might set up 10 workstations in one of three different network topologies (IBSS, BSS, ESS) and one of three different spatial distributions (circular, uniform, random). All of the ten computers spread out over three places will be outfitted with the same set of six IEEE technologies (802.11, 11a, 11b, 11g, 11n, and 11e). The scenarios can then be performed and the results evaluated. Each of the following quality of service statistics has to have a cumulative distribution function (CDF) distribution generated for it: End-to-end delay in packet transmission (sec), jitters (sec), throughput (in bits per second), and the percentage of lost packets. Table 2 can be used to implement the algorithms and calculations used by the system.

**Figure 4:**
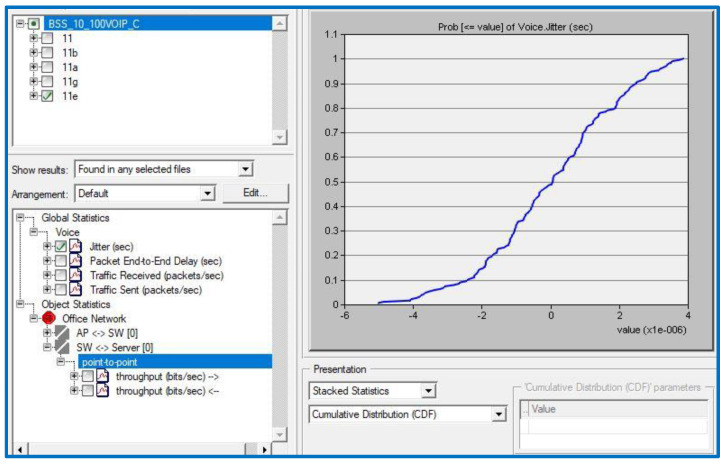
Jitter result of the scenario.

Initially, it is necessary to define the QPM, which is the value produced in the CDF for VoIP by implementing the necessary threshold (QoS parameter) for each performance criterion. For each QoS parameter (H = 1, M = 0.5, L = 0.1, and VL = 0), the QFM value will be calculated using QPM weighting (as determined by importance). Additionally, each project will incorporate the six WLAN physical layer technologies into six distinct scenarios (802.11, 11a, 11b, 11g, 11n, and 11e). When the six scenarios have run for 20 min, the effects of each QoS parameter will be evaluated in the same way. The 802.11e scenario is used for the following computations:

- Jitter:

Table 2 demonstrates that a jitter value of 0.04 s is a VoIP service threshold where QoS application importance is high. The QPM is 1, as seen by the outcome in Figure 4. To calculate the QFM for jitter, we simply multiply 1 by the importance coefficient of 1 to obtain 1.

**Figure 5:**
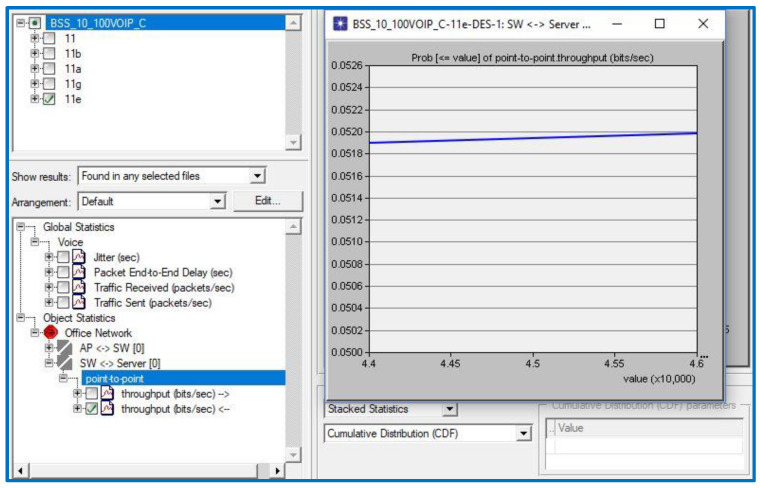
Throughput results of the scenario.

- Throughput:

As can be seen in Table 2, the VoIP throughput threshold value is 45 kbps when QoS application importance is set to medium. In Figure 5, we see that the QPM equals 0.052. To calculate the QFM, multiply 0.052 by its weight of 0.5, which is 0.0026 (0.5 is used because the throughput importance coefficient is medium (M = 0.5)).

The same technique is to derive QoS data for various WLAN technologies (11, 11n, 11a, 11b, and 11g). The QFM values for each WLAN standard will be aggregated to determine the AFMs for all technologies. Based on WLAN SFM technologies, as indicated in Table 4, a ranking list of these six methods is presented. When ranking the six WLAN technologies, the same method is used for both the uniform and random distributions. All QoS values (QPMs, QFMs, SFMs, and AFMs) for all six technologies related to both applications (best-effort and 50% VC) in IBSS and ESS networks across all three spatial distributions were calculated using the same system algorithms and applied to the BSS and ESS networks to determine the best performing WLAN technology (or technologies) across these two network configurations.

Table 4 summarizes the various settings that must be used in the OPNET Modeler to develop scenarios with mixed applications.

**Figure 6:**
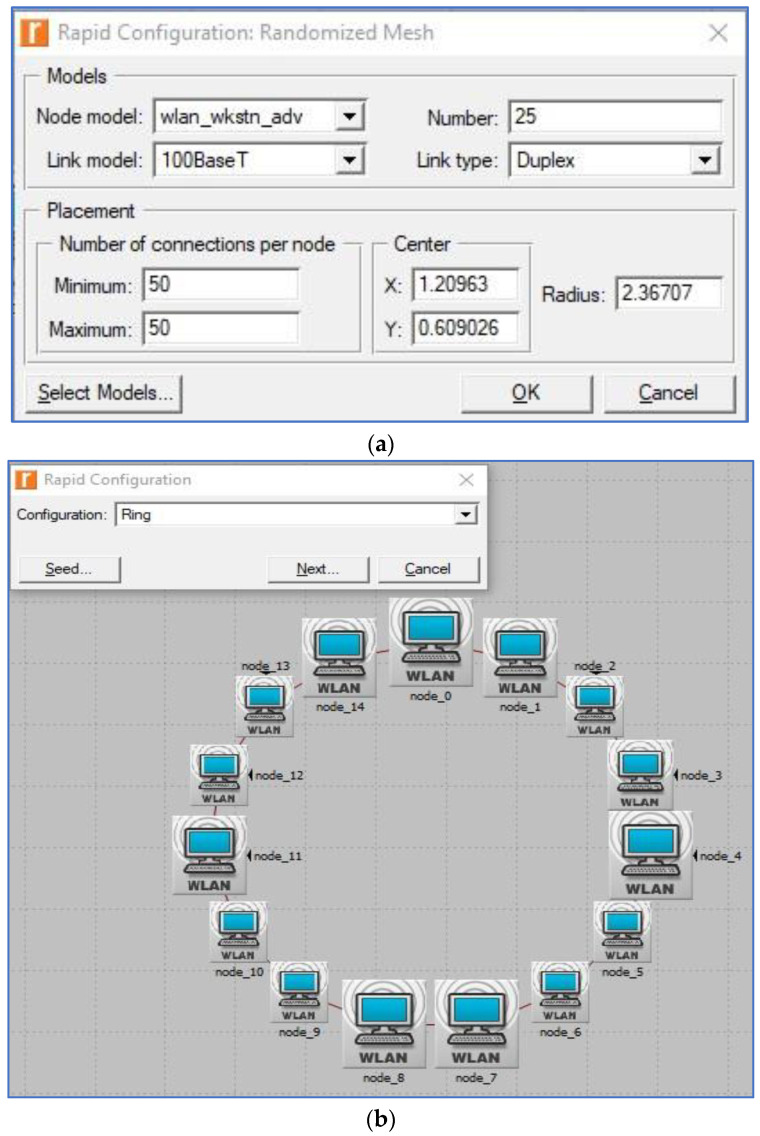

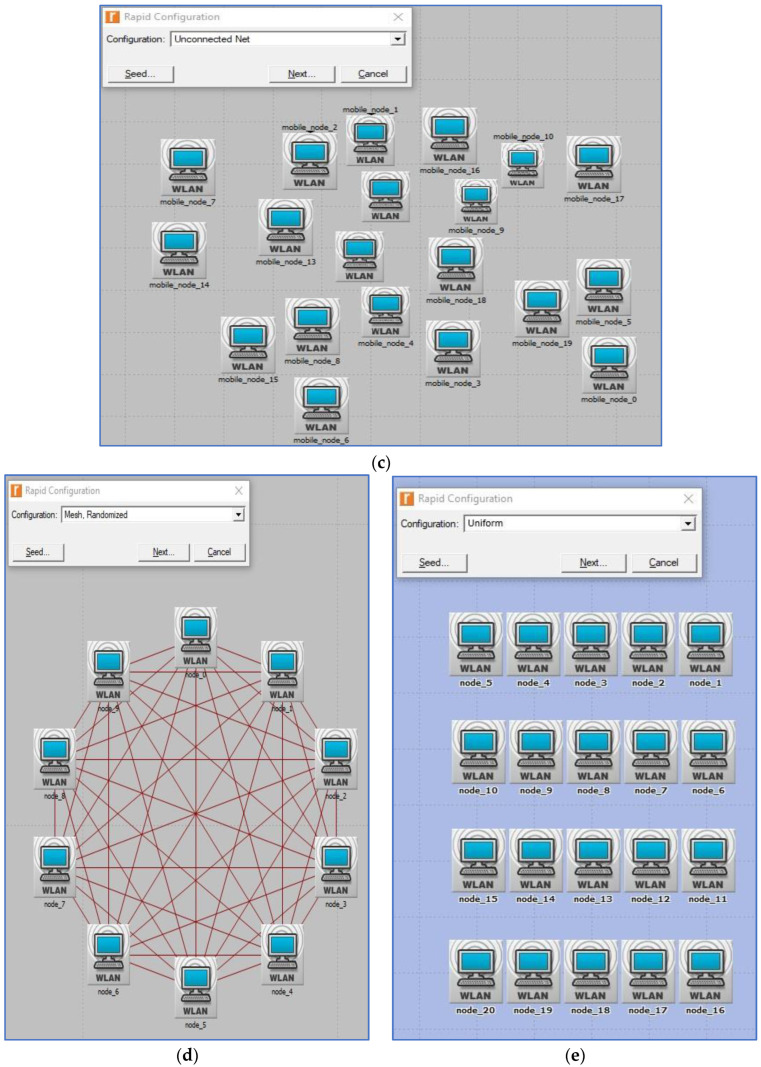
(**a**) Riverbed Rapid Configuration dialog box; (**b**) circular (ring) topology; (**c**) unconnected net (random) topology; (**d**) randomized mesh topology; (**e**) uniform topology (Riverbed, 2017).

We also analyzed how different spatial distributions (topologies) would affect the performance of each WLAN technology; however, instead of settling on one, we provided a range of options, such as uniform, circular, and random topologies. If a school or university needed to set up a lab, we figured that the nodes would be distributed in one of these three topologies. If a business requires a meeting or videoconferencing space with many computers, the same procedure will be followed to accommodate its needs. The devices have been distributed in every feasible way, including in a circle, uniformly, and randomly. Using Rapid Configuration, the Riverbed Platform library can generate a user-specified distribution from its C or C++ source code, as seen in Figure 6.

## 4. Performance Evaluation

In this section, we will examine the outcomes by making use of a wide array of application strategies. For each best-effort application (HTTP, FTP, and E-mail), as well as real-time applications (VoIP and Video Conferencing), IEEE 802.11 technologies were assessed in terms of three different spatial distributions: circular, random, and uniform distributions. In order to be more particular, we looked at how well the applications performed in circular, random, and uniform settings. The purpose of this study is to establish which network architecture is most suited for each of the five distinct mixed-use case studies discussed in this paper. This was accomplished by gathering information from a variety of sources. There are six possible technology rankings for IEEE 802.11, namely 802.11a, 11b, 11g, 11e, and 11n, each of which is designed for a certain combination of applications operating in a particular environment. According to the rankings of IEEE 802.11 technologies, the algorithm that has been suggested here would have the highest quality services in addition to the best overall network efficiency. In light of these attributes, the algorithm that has been provided would result in the highest possible level of service quality and the most effective overall network. Two distinct situations involving mixed applications were assessed and analyzed regarding a variety of parameters, which encompassed everything from location to the number of nodes to the architecture of the network as a whole. OPNET was used as an implementation tool, in particular Riverbed Modeler 17.5 [42].

Here, we want to clarify that in our minimal jitter- and greater throughput-centric critical investigation of a range of networking technologies for smart environments, two types of applications were used: best-effort applications and real-time applications. Three different network architectures were considered with a range of communication protocols for making prioritization decisions that focused on minimal jitter and greater throughput. Standard protocol settings and predefined similar traffic loads were applied in the implementation for better clarity in terms of jitter and throughput performance in the considered network scenarios and communication protocols:

1. Real-time applications consisting of 50% VoIP and 50% VC (Voice over Internet Protocol and Video Conferencing): All scenarios in this case study were solely focused on investigating and analyzing the mixtures in real-time applications. This phrase has been abbreviated to “50VC” for the sake of readability.
2. The best-effort applications should comprise 40% HTTP, 30% FTP, and 30% E-mail. The best-effort application mixtures tested here form the basis of all configurations used in this case study. “Best-effort” has been condensed in order to make the sentence more readable. A flowchart and a bar chart are included in the format of the results for every set of nodes and every mixed application, respectively. A flowchart has been used to determine the number of nodes, the network topologies, the spatial distributions, and the WLAN technologies that are being utilized.

**Figure 7:**
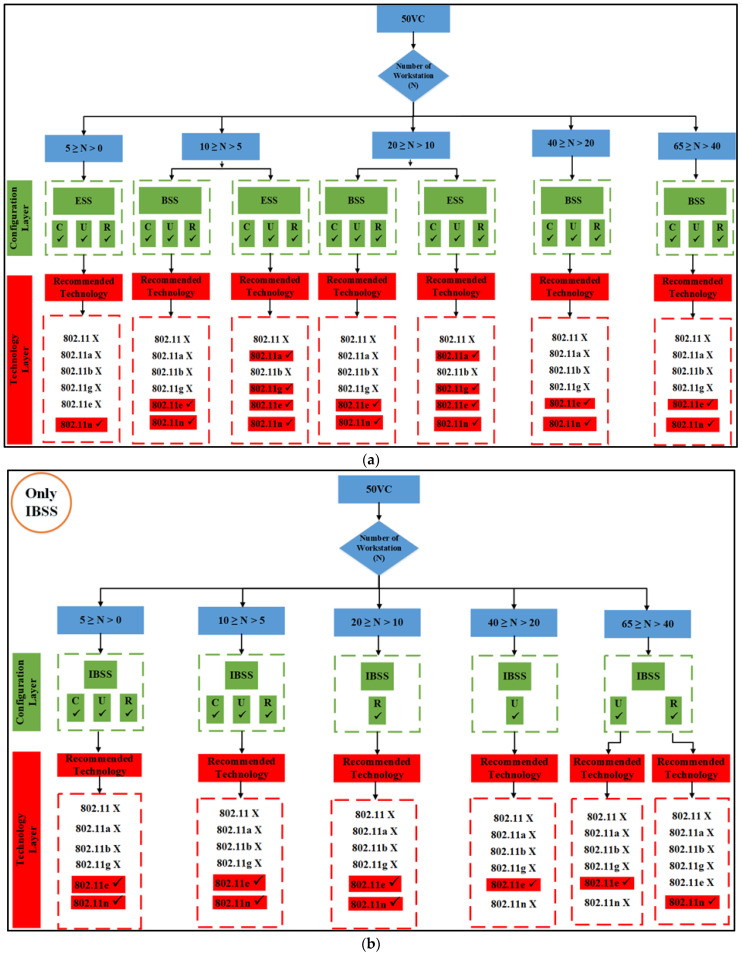
The proposed 50% VC algorithm. (**a**) BSS and ESS; (**b**) only IBSS.

The results obtained in mixed applications are referred to as the Scenario Fitness Metrics (SFMs). Because the outcomes depend on the presence of the access point, the tables of outcomes are displayed (interpreted), which will be demonstrated later for each application in two flowcharts: the IBSS chart and the generic flowchart. The very last thing that needs to be accomplished is that the technology that has the optimum performance for each individual case study (network configuration) needs to be highlighted, as well as the optimal choice for each group of nodes:

- If there is at least one access point available, the suggested method is implemented in the manner depicted in Figure 1, and the flowchart results are displayed in Figure 7a, and subsequently. This is true for the BSS architecture layer, as well as the ESS architecture layer.
- The method proposed in Figure 1, as well as the algorithms shown in Figure 7b, and subsequently for the findings of the IBSS network, can be utilized even if the network is set up without an access point.

### 4.1. A Mixture of Applications Working in Real Time (50% VoIP and 50% VC)

**Figure 8:**
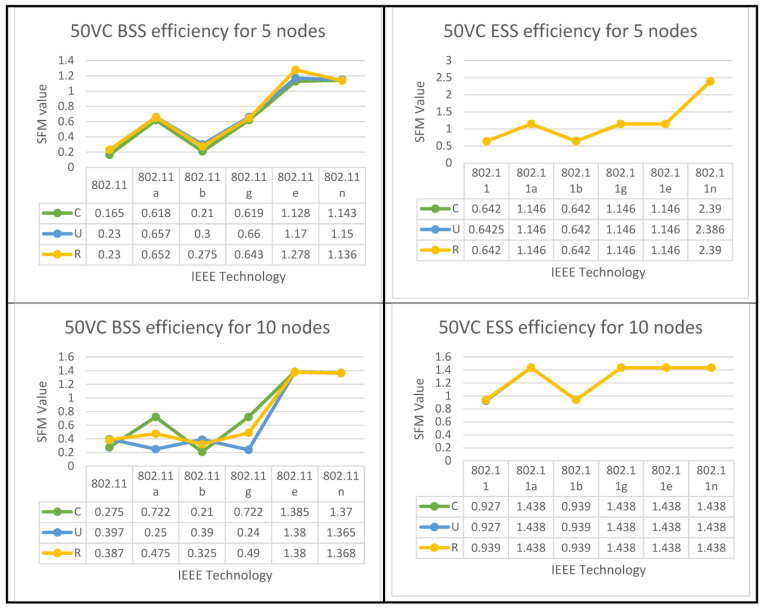

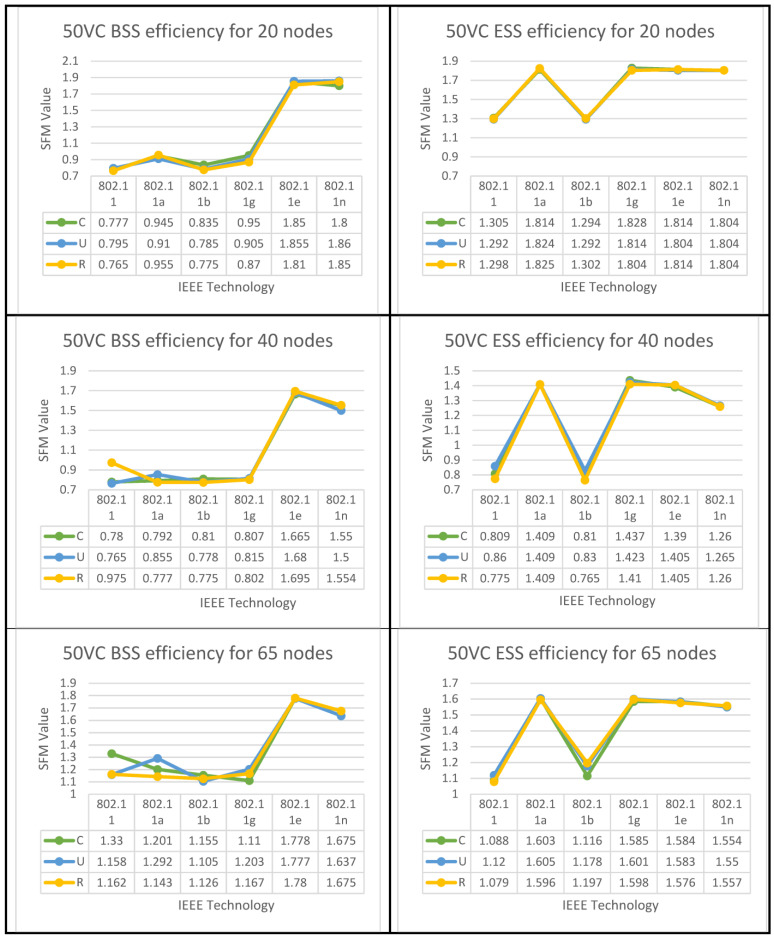
BSS & ESS Performance Optimization for (50%VC algorithm).

**Figure 9:**
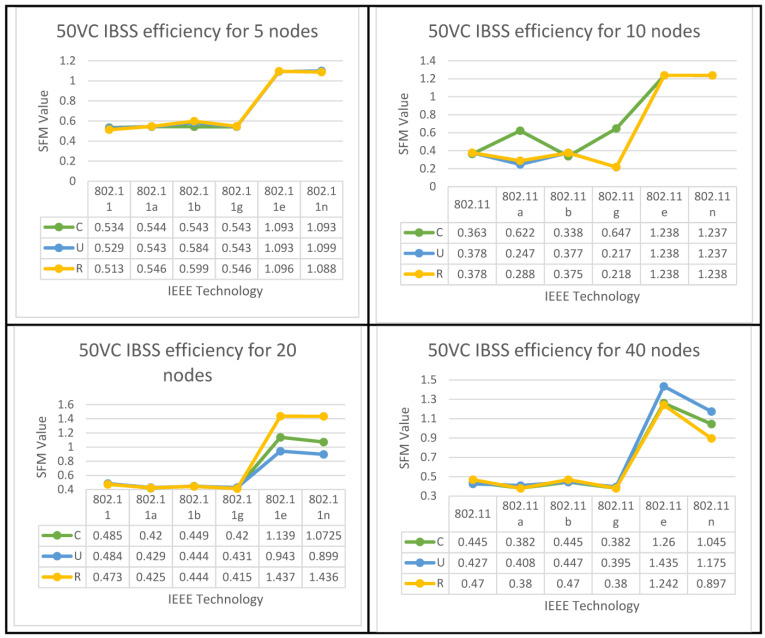

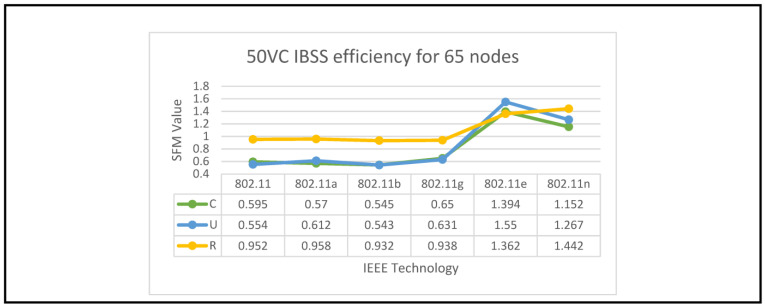
IBSS Performance Optimization for (50%VC algorithm).

**Figure 10:**
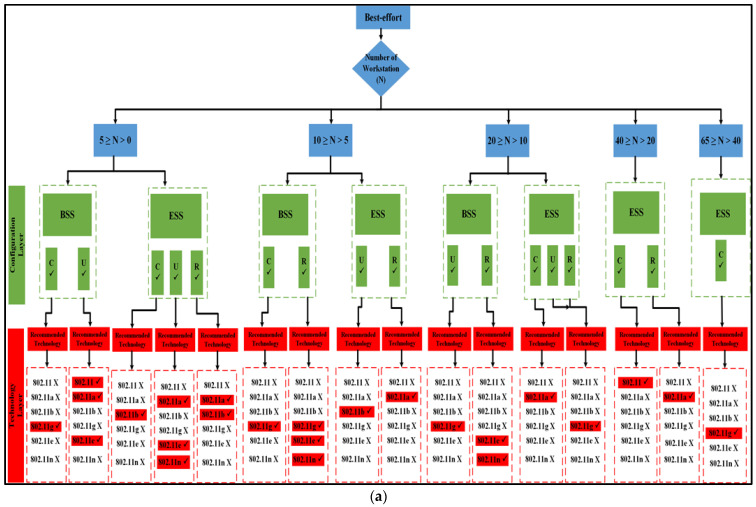

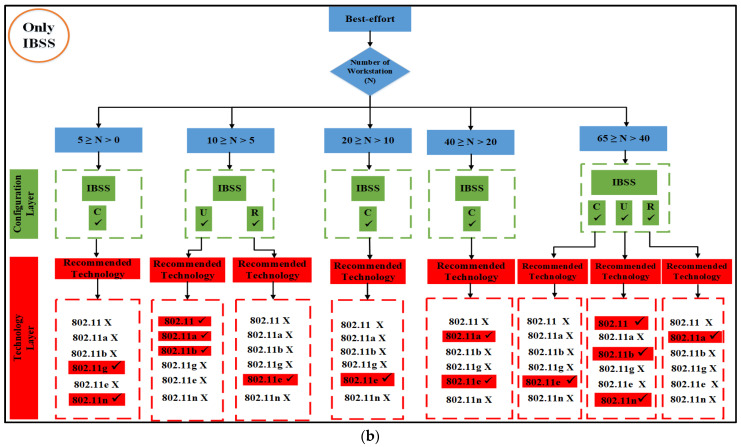
Proposed best-effort algorithm. (**a**) BSS and ESS; (**b**) only IBSS.

The algorithms for both set of results are displayed below in Figure 8, Figure 9 and Figure 10 for all six IEEE 802.11 technologies across all 65 nodes in the case study with three different geo-graphical deployments involving 50% VoIP and 50% VC. These figures cover the entirety of the case study and apply to all six IEEE 802.11 technologies. In addition, a case study that was conducted consisted of calls that were split evenly between VoIP and traditional videoconferencing (C, U, R). Both technology 11e and technology 11n produced nearly optimal performance for all spatial distributions, which is consistent with the case of IBSS, with the exception that the uniform and random distributions are dominant for medium and larger nodes. Both of these technologies are consistent with the case of IBSS (20–65). The research indicates that the performance of ESS and BSS for each of the five groups of nodes is virtually the same. In addition, the findings indicate that both approaches generate performance that is nearly optimal across the board for spatial distributions.

IBSS reaches its maximum performance potential on both 802.11e and 11n when the client builds a network with a limited number of nodes. This holds true for both of these standards, where 5 ≥ N > 0. It turns out that the only technologies that perform adequately in the second and third groups of space distribution are IEEE 802.11e and 11n. This is the case for both of these groups.

Following the flowchart for the IBSS led to the discovery of this information. 802.11n will deliver the best performance in the fifth category if the configuration is randomized; however, if the setup is uniform, 802.11e will be the best option for categories four and five in the IBSS results. Figure 9 provides a visual representation of this point.

### 4.2. A Mixture of Applications Made with Best Effort (40% HTTP, 30% E-Mail, and 30% FTP)

**Figure 11:**
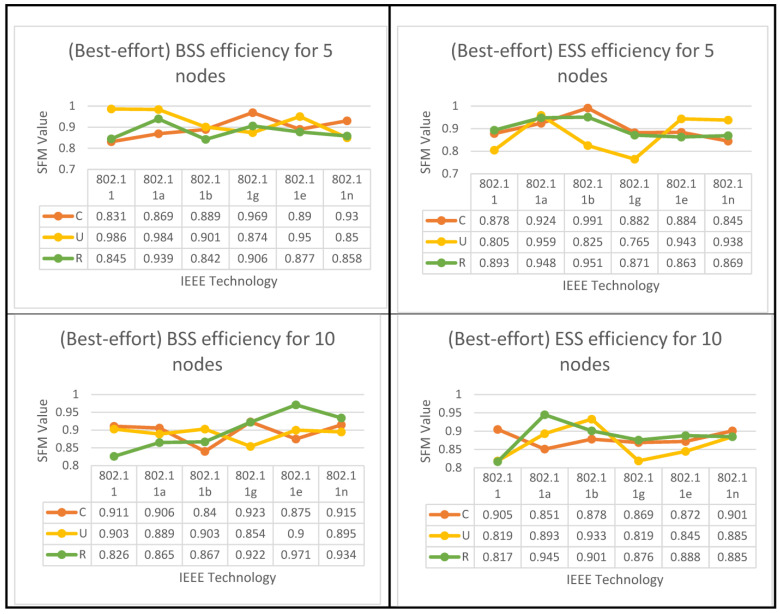

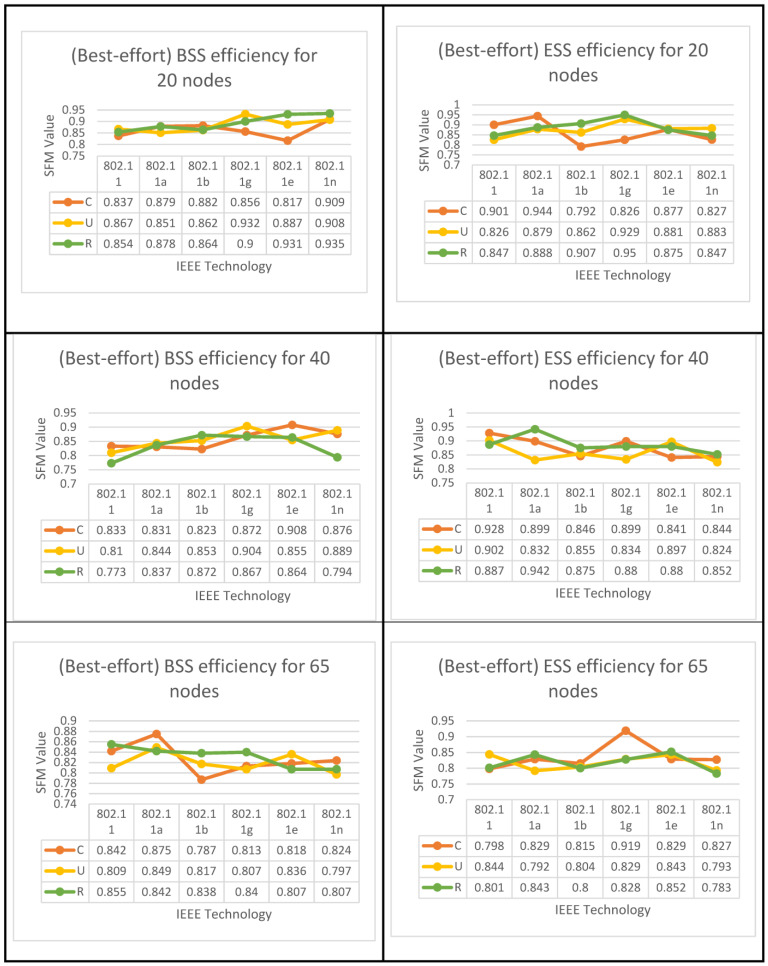
BSS and ESS performance optimization for the best-effort algorithm.

**Figure 12:**
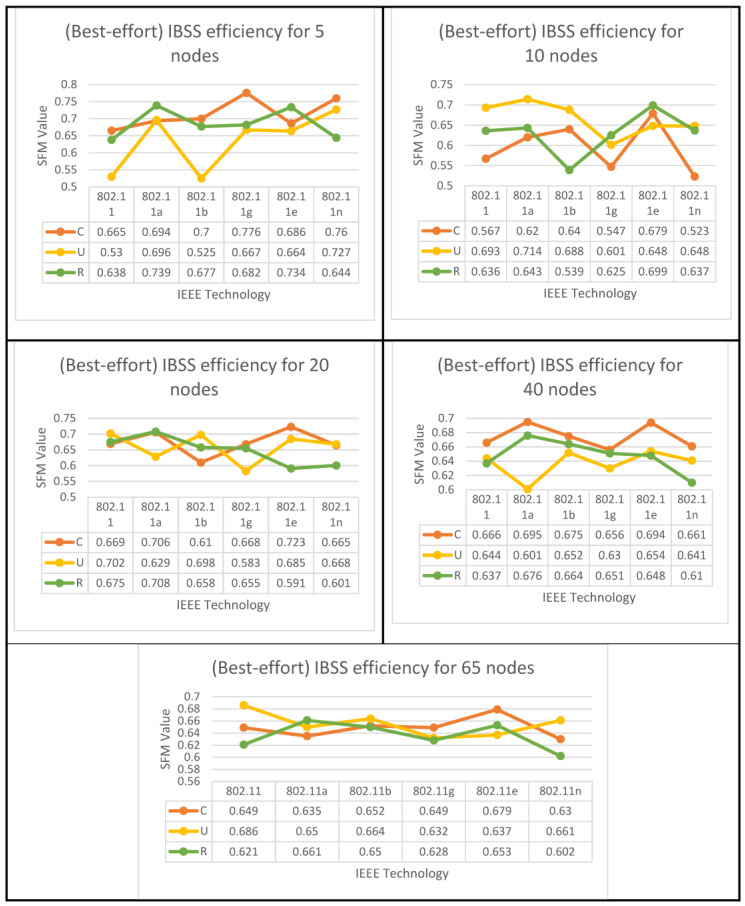
IBSS performance optimization for best-effort applications.

The results of both algorithms are depicted in Figure 10, Figure 11 and Figure 12 for all nodes that participated in the best-effort case study and used all six WLANs. Three spatial configurations using IEEE 802.11 standards are examined. As can be observed in Figure 10a and Figure 11, both network topologies, BSS and ESS, are able to function in small and medium-sized networks (1–20), with 11g, 11e, and 11n technologies providing the highest level of performance. In the first group, where 5 ≥ N > 0, there is a variety of options provided by BSS and ESS. Only a circular network layout benefits from the use of IEEE 802.11g technology in BSS architecture. As an added note, IEEE 802.11, 11a, and 11e technologies only function properly in a standard setting. ESS, on the other hand, provides a number of alternatives. The only situations where IEEE 802.11b is considered the best option are those with a circular or random distribution of problems. Figure 11 and Figure 12 show that the best user output is achieved with a circular configuration of IEEE 802.11g and 11n technologies, respectively, while the best performance is achieved with a uniform configuration of IEEE 802.11e and 11n technologies. In the second range, 10 ≥ N > 5, BSS and ESS have a wide variety of options to choose from. For BSS architecture, IEEE 802.11g technology performs best with a circular network layout.

In addition, the performance of the IEEE 802.11g, 11e, and 11n technologies was outstanding, even when the configuration was performed in a haphazard manner. On the other hand, ESS provides users with a variety of choices from which to select. It has been demonstrated that the optimal choice is IEEE 802.11b when the network is configured in a consistent manner. Furthermore, as can be seen in Figure 11, the optimal configuration for IEEE 802.11a is one in which the settings are completely arbitrary, which results in the best performance. According to the findings of the IBSS network, the optimal results for the client can be achieved with a circular deployment of 802.11g and 11n, which is depicted as an example in Figure 12.

In the third group, where 20 ≥ N > 10, both BSS and ESS offer a wide range of different choices to their customers. Only when the network is constructed consistently do IEEE 802.11g technologies perform to their full potential in BSS architectures. In addition, the optimal performance of IEEE 802.11e and 11n technologies can only be achieved when the configuration is performed in a randomized fashion. Nonetheless, the ESS architecture provides a variety of options to select from. Only in a network that is a completely closed loop will IEEE 802.11a become the superior choice. In addition, the performance of IEEE 802.11g is excellent, regardless of whether the configuration is uniform or arbitrary, whereas the results of the IBSS architecture show that IEEE 802.11e has the best performance when it comes to circular distribution.

According to the standard flowchart, the ESS architecture achieves its highest level of performance in the fourth and fifth categories. The client has the option of choosing between two different sets of nodes for the fourth set based on the data that are presented in Figure 11. The 802.11 standard is the way to go if you only plan on arranging your network in a circular fashion as part of your deployment.

The other alternative is to employ 802.11a technology with a haphazard setup. Using 802.11g technology in a circular configuration is ideal for the fifth set of nodes. Figure 12 shows that if only a circular network is designed for an IBSS, either 802.11a or 11e would be the best technology to use. The 802.11e standard is recommended for use in the fifth category if the network is to be set up in a circular configuration, while the 802.11, 11b, or 11n standards are more appropriate for use in a network set up in a uniform fashion. However, if 802.11a is configured arbitrarily, it is the best choice.

### 4.3. Mixed Service Scenario Layer Configuration

This section describes how to configure the configuration and technology layers of a system using the system’s algorithms and their corresponding mathematical models (equations), with examples of how this is achieved in the context of mixed-service use cases. A university has requested a networking lab, which will have 40 machines due to the 40 students enrolled. The university estimates that, at any given time, traffic will be split between best-effort services, with 40% allocated to HTTP, 30% allocated to E-mail browsing, and 30% allocated to file sharing and transfer. The university makes the call after determining how much time will be spent on the internet for things such as HTTP traffic, E-mail, and file transfer. The optimal IEEE technology and network architecture, as well as the optimal spatial distribution of these 40 machines, should be provided by our algorithm and mathematical equations.

**Figure 13:**
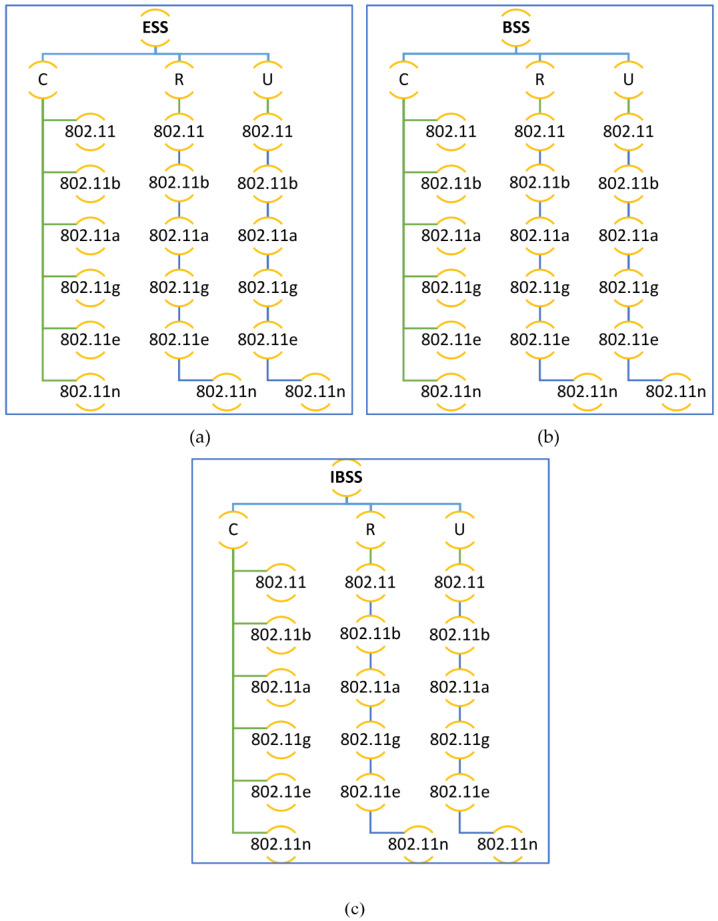
Project scenarios for all three network configurations: (**a**) BSS, (**b**) ESS, and (**c**) IBSS.

Forty machines will initially be set up in 54 configurations due to the fact that there are three major projects to be built for each of the three main network configurations (BSS, ESS, IBSS), and all of these must take into account the spatial distributions of the workstations (circular, uniform, random). For each possible configuration of spatial distributions for the six WLAN technologies, six separate scenarios are developed (802.11, 11a, 11b, 11g, 11e, and 11n). In addition, there are four applications represented here, with 40% of nodes running HTTP (16 machines), 30% running FTP (12 machines), and 30% running E-mail (12 machines). Figure 13 provides a graphical representation of the projects involved in the BSS, ESS, and IBSS scenarios.

In the second phase, depicted at the bottom of Figure 1, the system calculations and mathematical model are displayed. The CDF distribution for each application’s QoS metric and the QoS threshold values for those applications are used as inputs for the algorithm’s calculations. In Table 2, we can see the relative threshold values for each mixed-use application and the relative qualitative importance of each QoS parameter. Once the OPNET simulation scenarios have been run, a CDF distribution is generated for these QoS metric parameters. For each mixed application, we will calculate the underlying mathematical equations to understand how a given scenario has met the required performance metrics. This algorithm’s computations are explained, and the results of the three projects are analyzed using the following equations.

Table 5 reveals that among the three mixed services, HTTP accounts for 40%, FTP for 30%, and E-mail for 30%. Quantitative QoS parameters *n* for each type of mixed service will be calculated. First, the QoS parameters for HTTP 40% are used to calculate the QPM for both metric parameters across all six IEEE technologies (g). Next, the QFM is calculated across all six technologies using Equation (2). These two equations will be used to determine the QPM and QFM if throughput is used as TH, and the implementation of these equations and the methods for determining these quantities are described in detail in the sub-section above.

**Figure 14:**
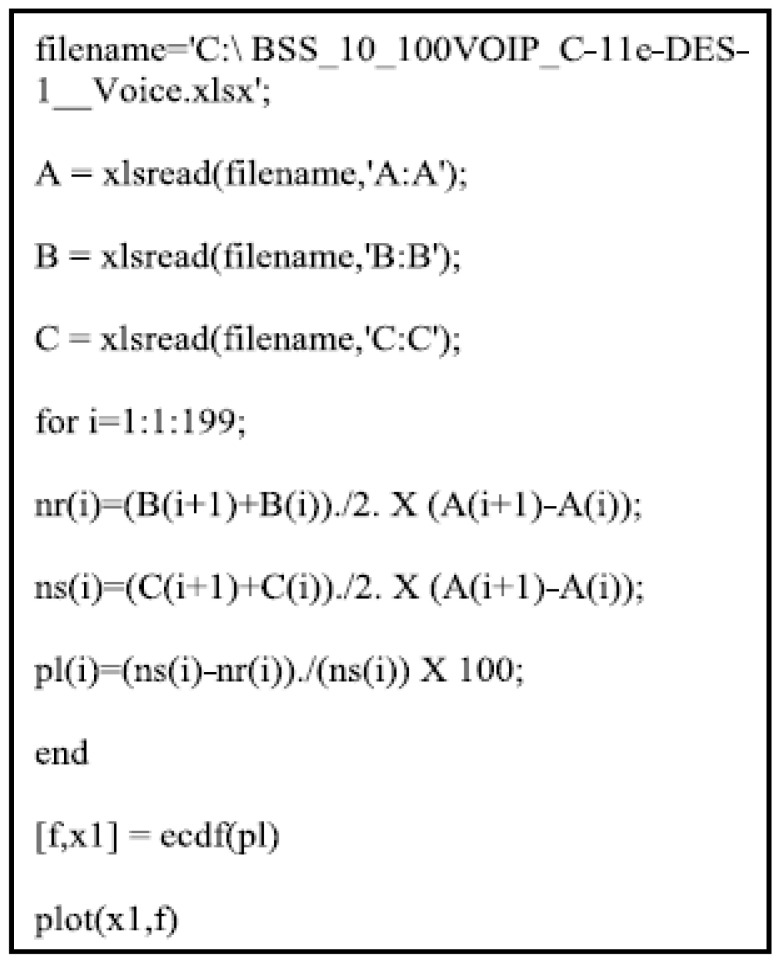
Evaluating packet loss through the utilization of MATLAB.

The percentage of packets lost ($\omega_{i}$) by an application on a given node i as of Equation (4) shows that, for the percentage of dropped data packets ki divided by the total number of dropped packets $\rho_{i}$ multiplied by 100%, a code has been written in MATLAB to determine this percentage for each of the three mixed-use cases, as shown in Figure 14. This technique is integrated with the OPNET Modeler to generate an accurate packet loss percentage for each unique combination of applications and IEEE technologies. The AFM for individual services and the SFM for combined services will be determined using Equation (3) and (5).

(5) $${\mathrm{SFM}}_{g}=\overset{4}{\underset{n=1}{\sum }}{\mathrm{AFM}}_{n}$$

The WLAN SFM values are used to rank these six technologies for the circular distribution of mixed services in BSS scenarios. When ranking the six WLAN technologies, the same method will be used for both the uniform and random distributions. For the ESS and IBSS network topologies, the system will apply algorithms and perform calculations to identify the top-performing WLAN technology in each topology and generate the QPMs, QFMs, AFMs, and SFMs for all QoS parameters for all six technologies with respect to each mixed service in this scenario’s projects across all three spatial distributions.

Table 6 displays the relative rankings of all WLAN technologies for each of the three geographical distributions of the best-effort mixed services.

## 5. Conclusions

We have expanded our previous work to the implementation of Internet applications as a stand-alone service that includes mixed applications. Different services are executed and configured at different rates depending on the circumstances. Additionally, new nodes and IEEE technologies were included. In this body of work, a novel approach to analyzing network performance was developed. The results of 50% VC applications show that it is only preferable to use the BSS network with a high number of workstations/nodes for all three spatial distributions. This is due to high packet loss and delays that might appear in the network owing to the increase in the number of workstations. Interestingly, ESS networks are suitable for best-effort services for a large number of nodes. Furthermore, IBSS networks worked efficiently with 802.11e and 802.11n technologies for almost all selected numbers of nodes for 50% VC when they were configured randomly or uniformly in medium-sized networks, whereas for best-effort services the performance of different technologies was based on the size of the network, with the circular distribution shown to be the dominant distribution.

The next step for this study is to apply the same methods developed here to other real-time and best-effort qualities, as well as upcoming ones, in order to determine the most appropriate IEEE standard for use with different kinds of application services. As a result of the increased network size and the incorporation of new network protocols, we intend to propose and implement new applications for the resulting service mix. The Internet of Things (IoT), which has expanded from simple machine-to-machine (M2M) communication, is just one example of the many promising directions in which researchers could go next. M2M communications are useful for businesses because they allow computers to be connected to the cloud, devices to be controlled remotely, and data to be collected. The connectivity enabled by M2M communication is the backbone of the Internet of Things. To continue, it is expected that Machine Learning (ML) algorithms and Artificial Intelligence (AI) will serve as the backbone of many different kinds of technologies and applications. Answers and forecasts from ML depend on the data available from the network. Finally, wireless networks have become increasingly popular, with the release of Wi-Fi 6 (IEEE 802.11ax) and Wi-Fi 7 (IEEE 802.11be) attracting an ever-increasing number of users. As a result, digital information consumption across all mediums has increased dramatically.

## Figures and Tables

**Table 1:** Outcomes comparing the proposed method to existing mixed-algorithm solutions.

Reference	Approach	Parameters for Measuring Quality of Service	The Number of Nodes in the Network	Architectural Components of a Network	Technology Developed by the IEEE	Modeling Simulation	Limitation
[20]	This article discussed how various performance assessment procedures might affect ad hoc networks and their effectiveness. Here, using NS2, the authors compare the performance of TCP, UDP, and SCTP over a range of metrics.	Jitter End-to-end delay Throughput Packet loss	10	IBSS	802.11	NS2	It used a limited number of nodes and only one IEEE standard technology in its architecture.
[18]	A wireless fiber architecture that combines a 5G WLAN with a 10G passive optical network (XGPON) was examined in this paper (IEEE 802.11ac). Both technologies have their advantages and disadvantages in regard to satisfying the quality of service requirements of paying customers.	Bandwidth Fairness End-to-End delay	8	BSS	802.11ac	OMNET++	The number of nodes that were utilized is not particularly high, and the network architecture and IEEE technology utilized were both singular.
[19]	In order to provide high QoS for various multimedia (video, voice, and FTP) services that customers need, the standard EDCA effective service differentiation structure is activated and evaluated (particularly critical or time-sensitive services).	Delay Throughput	3, 9, and 18	BSS	802.11n	OPNET	It only makes use of two QoS parameters, whereas the technology only made use of one.
[14]	In this study, an OPNET simulation was utilized to examine how different QoS methods affect the performance and capacity of a VoIP network. How high VoIP technology may go while still providing quality that meets standards was also explored.	Jitter Delay Throughput	5	ESS	802.11	OPNET	There was only one type of network architecture deployed, and the number of nodes was relatively low.
[13]	Taking into account all conceivable QoS methods, this research measures and assesses the behavior of web-based apps during a vertical handover between 802.16e and 802.11e technologies. OPNET Modeler was used to carry out the scenario evaluation. Used software included E-mail and web traffic, both of which were dynamic (HTTP + database).	HTTP load page delay Mail download and upload delay TCP delay DB query delay	2	BSS WiMAX	802.16e 802.11e	OPNET	The study only involves two nodes and two IEEE technologies.
[21]	In terms of network performance, the impact of the RTS and fragmentation thresholds was assessed. Additionally, different MAC access methods were used to assess the network’s speed, and the findings were compared to industry norms.	Jitter End-to-end delay Throughput	10	IBSS	802.11e 802.11g	OPNET	This research used only IEEE 802.11e/g and a small network of 10 nodes.
[20]	Exploration of how well the XG-PON and EDCA optimized network design deals with rapid growth in real-time traffic as a result of current global IP traffic distribution.	End-to-end delay Jitter Fairness Throughput	16	BSS	802.11n	NS3	There was only one technology and one type of BSS network architecture used in this research.
[15]	The robust performance of the OPNET-based communication IP network simulation model enabled the modeling of real-world network scenarios, the incorporation of performance specifications for the operation of existing equipment, and the provision of a versatile graphical environment and design for network communication.	Link data rate Throughput Delay	NA	ESS	802.11	OPNET	There was only one IEEE technology discussed, and the number of nodes used was not provided.
[16]	This article built simulations of different standard smart meter networks using evaluation metrics. Databases could be queried and files could be uploaded using both wired and wireless communications during typical data transmission and DDoS attacks on the network.	FTP request response from the server HTTP request received by the server	20	BSS	802.11	OPNET	The technology employed is old and outdated, and modern advancements are not even mentioned.
[12]	This technique evaluated the impact of jitter and delays in the network with regard to improving MAC layer QoS in a Wi-Fi downlink. According to the results of the simulations, the new classification of time-critical traffic access improved efficiency and led the way for the widespread deployment of time-sensitive networking and Wi-Fi systems in a variety of manufacturing settings.	Delay Jitter	20	BSS	802.11ac	Monte Carlo simulation	Two quality of service parameters and a single network topology were employed.
[11]	Review and evaluation of IEEE 802.11n random topology WLAN multimedia services are the focus of this research. The standard’s impact on the network’s output was explained by the optimized structure that included the necessary spatial stream of features at the MAC layer.	Attempts at retransmission and data loss Throughput Delay	3, 9, and 18	BSS	802.11n	OPNET	As well as only employing a single network architecture and a single IEEE 802.11 technology, the nodes used did not extend to medium or large networks.
Proposed study	Analyzed the best protocol and network architecture based on mixed application metrics for various IEEE 802.11 technologies.	Packet loss Jitter Throughput Delay	1–65	BSS ESS IBSS	802.11 802.11a 802.11b 802.11g 802.11e 802.11n	OPNET	

**Table 2:** Importance of QoS metrics for online applications.

Application	Importance (I) and Threshold (T)	Jitter (sec)	Packet Loss Rate (%)	Delay (sec)	Throughput (kbps)
E-mail	I	Very Low	Low	Low	Low
	T	0	10	1	30
HTTP	I	Very Low	Low	Medium	Low
	T	0	10	1	30
VC	I	High	Medium	High	High
	T	0.03	1	0.15	250
FTP	I	Very Low	High	Low	Medium
	T	0	5	1	45
VoIP	I	High	Low	High	Medium
	T	0.04	5	0.15	45

**Table 3:** Data rates of IEEE 802.11 standards.

**IEEE Standards**	802.11	802.11a	802.11b	802.11g	802.11e	802.11n
**Data rate (Mbps)**	2	6	2	6	11	(base)/60 (max)

**Table 4:** Settings and parameters used in typical simulations.

System Settings			
1	Profile start time (sec)	60	
2	Simulation time (min)	20	
3	Value Per Statistic	200	
4	IP Routing	EIGRP Enable	
5	VC	Parameters	Values
		Frame Interarrival Time Information	10–15 frames/sec
		Symbolic Destination Name	Video Destination
		Frame Size Information (bytes)	128 × 120/128 × 240 pixels
		Type of service (TOS)	Interactive multimedia
6	VoIP	Parameters	Values
		Voice frame per packet	1
		Application	Voice
		Codec	G.711
		Compression and Decompression delay	0.02 sec
		Types of service (TOS)	Interactive voice
7	HTTP	Parameters	Values
		HTTP Specification	HTTP 1.1
		Page Interval Time (sec)	Exponential (60)
		Types of service (TOS)	Best Effort
8	FTP	Parameters	Values
		Command Mix (Get/Total)	50%
		Inter-Request Time (sec)	Exponential (360)
		File Size (bytes)	50,000
		Types of service (TOS)	Best Effort
9	Email	Parameters	Values
		Send Interarrival Time (sec)	Exponential (360)
		Receive Interarrival Time (sec)	Exponential (360)
		E-Mail Size (bytes)	20,000
		Symbolic Server Name	Email Server
		Types of service (TOS)	Best Effort

**Table 5:** Service mix (40% HTTP, 30% FTP, and 30% E-mail).

	Technology	802.11		802.11b		802.11a		802.11g		802.11e		802.11n	
Application													
Email 30%	*PR*	*QFM_PR_*		*QFM_PR_*		*QFM_PR_*		*QFM_PR_*		*QFM_PR_*		*QFM_PR_*	
	*TH*	*QFM_TH_*	*QFM_TH_*	*QFM_TH_*	*QFM_TH_*	*QFM_TH_*	*QFM_TH_*	*QFM_TH_*	*QFM_TH_*	*QFM_TH_*	*QFM_TH_*	*QFM_TH_*	*QFM_TH_*
	*PL*	*QFM_PL_*		*QFM_PL_*		*QFM_PL_*		*QFM_PL_*		*QFM_PL_*		*QFM_PL_*	
	1	Sum (*QFM_J_* + *QFM_D_* + *QFM_TH_* + *QFM_PL_*) × 40%		*AFM_11b_*		*AFM_11a_*		*AFM_11g_*		*AFM_11e_*		*AFM_11n_*	
HTTP 40%	*PR*	*QFM_PR_*		*QFM_PR_*		*QFM_PR_*		*QFM_PR_*		*QFM_PR_*		*QFM_PR_*	
	*TH*	*QFM_TH_*	*QFM_TH_*	*QFM_TH_*	*QFM_TH_*	*QFM_TH_*	*QFM_TH_*	*QFM_TH_*	*QFM_TH_*	*QFM_TH_*	*QFM_TH_*	*QFM_TH_*	*QFM_TH_*
	*PL*	*QFM_PL_*		*QFM_PL_*		*QFM_PL_*		*QFM_PL_*		*QFM_PL_*		*QFM_PL_*	
	2	Sum (*QFM_PR_* + *QFM_TH_* + *QFM_PL_*) × 40%		*AFM_11b_*		*AFM_11a_*		*AFM_11g_*		*AFM_11e_*		*AFM_11n_*	
FTP 30%	*DR*	*QFM_DR_*		*QFM_DR_*		*QFM_DR_*		*QFM_DR_*		*QFM_DR_*		*QFM_DR_*	
	*TH*	*QFM_TH_*	*QFM_TH_*	*QFM_TH_*	*QFM_TH_*	*QFM_TH_*	*QFM_TH_*	*QFM_TH_*	*QFM_TH_*	*QFM_TH_*	*QFM_TH_*	*QFM_TH_*	*QFM_TH_*
	*PL*	*QFM_PL_*		*QFM_PL_*		*QFM_PL_*		*QFM_PL_*		*QFM_PL_*		*QFM_PL_*	
	3	Sum (*QFM_DR_* + *QFM_TH_* + *QFM_PL_*) × 30%		*AFM_11b_*		*AFM_11a_*		*AFM_11g_*		*AFM_11e_*		*AFM_11n_*	
SFM		1 + 2 + 3		*SFM_11b_*		*SFM_11a_*		*SFM_11g_*		*SFM_11e_*		*SFM_11n_*	
Rank		IEEE technology		IEEE technology		IEEE technology		IEEE technology		IEEE technology		IEEE technology	

**Table 6:** Outcomes for best-effort mixed BSS, IBSS, and ESS services across 40 workstations.

	Application	BSS			IBSS			ESS		
Technology		C	U	R	C	U	R	C	U	R
802.11		*SFM_11_*	*SFM_11_*	*SFM_11_*	*SFM_11_*	*SFM_11_*	*SFM_11_*	*SFM_11_*	*SFM_11_*	*SFM_11_*
802.11a		*SFM_11a_*	*SFM_11a_*	*SFM_11a_*	*SFM_11a_*	*SFM_11a_*	*SFM_11a_*	*SFM_11a_*	*SFM_11a_*	*SFM_11a_*
802.11b		*SFM_11b_*	*SFM_11b_*	*SFM_11b_*	*SFM_11b_*	*SFM_11b_*	*SFM_11b_*	*SFM_11b_*	*SFM_11b_*	*SFM_11b_*
802.11g		*SFM_11g_*	*SFM_11g_*	*SFM_11g_*	*SFM_11g_*	*SFM_11g_*	*SFM_11g_*	*SFM_11g_*	*SFM_11g_*	*SFM_11g_*
802.11e		*SFM_11e_*	*SFM_11e_*	*SFM_11e_*	*SFM_11e_*	*SFM_11e_*	*SFM_11e_*	*SFM_11e_*	*SFM_11e_*	*SFM_11e_*
802.11n		*SFM_11n_*	*SFM_11n_*	*SFM_11n_*	*SFM_11n_*	*SFM_11n_*	*SFM_11n_*	*SFM_11n_*	*SFM_11n_*	*SFM_11n_*

## Data Availability

Research data will be available on individual requests to the corresponding author considering collaboration possibilities with the researcher or research team and with restrictions that the data will be used only for further research in the related literature progress.
